# Combination of organic acids benzoate, butyrate, caprylate, and sorbate provides a novel antibiotics-independent treatment option in the combat of acute campylobacteriosis

**DOI:** 10.3389/fmicb.2023.1128500

**Published:** 2023-03-15

**Authors:** Ke Du, Minnja S. Foote, Soraya Mousavi, Agnes Buczkowski, Sebastian Schmidt, Elisa Peh, Sophie Kittler, Stefan Bereswill, Markus M. Heimesaat

**Affiliations:** ^1^Gastrointestinal Microbiology Research Group, Institute of Microbiology, Infectious Diseases and Immunology, Charité – University Medicine Berlin, Corporate Member of Freie Universität Berlin, Humboldt-Universität zu Berlin, Berlin Institute of Health, Berlin, Germany; ^2^Hofmann & Sommer GmbH & Co. KG, Büro Berlin, Berlin, Germany; ^3^Institute for Food Quality and Food Safety, University of Veterinary Medicine Hannover, Hannover, Germany

**Keywords:** benzoic acid, butyric acid, caprylic acid, sorbic acid, acute *Campylobacter jejuni* induced enterocolitis, host–pathogen interaction, organic acids, placebo controlled intervention study

## Abstract

**Introduction:**

The food-borne Gram-negative bacterial pathogen *Campylobacter jejuni* may cause the acute enterocolitis syndrome campylobacteriosis in infected humans. Given that human *C. jejuni* infections are rising globally which hold also true for resistance rates against antibiotic compounds such as macrolides and fluoroquinolones frequently prescribed for the treatment of severe infectious enteritis, novel antibiotics-independent therapeutic strategies are needed. Distinct organic acids are well known for their health-beneficial including anti-microbial and immunomodulatory properties. In our present study, we investigated potential pathogen-lowering and anti-inflammatory effects of benzoic acid, butyric acid, caprylic acid, and sorbic acid either alone or in combination during acute murine campylobacteriosis.

**Methods:**

Therefore, secondary abiotic IL-10^–/–^ mice were perorally infected with *C. jejuni* strain 81–176 and subjected to a 4-day-course of respective organic acid treatment.

**Results and discussion:**

On day 6 post-infection, mice from the combination cohort displayed slightly lower pathogen loads in the duodenum, but neither in the stomach, ileum nor large intestine. Remarkably, the clinical outcome of *C. jejuni* induced acute enterocolitis was significantly improved after combined organic acid treatment when compared to the placebo control group. In support, the combinatory organic acid treatment dampened both, macroscopic and microscopic inflammatory sequelae of *C. jejuni* infection as indicated by less colonic shrinkage and less pronounced histopathological including apoptotic epithelial cell changes in the colon on day 6 post-infection. Furthermore, mice from the combination as compared to placebo cohort exhibited lower numbers of innate and adaptive immune cells such as neutrophilic granulocytes, macrophages, monocytes, and T lymphocytes in their colonic mucosa and lamina propria, respectively, which also held true for pro-inflammatory cytokine secretion in the large intestines and mesenteric lymph nodes. Notably, the anti-inflammatory effects were not restricted to the intestinal tract, but could also be observed systemically given pro-inflammatory mediator concentrations in *C. jejuni* infected mice from the combination organic acid treatment that were comparable to basal values. In conclusion, our *in vivo* study provides first evidence that an oral application of distinct organic acids in combination exhibits pronounced anti-inflammatory effects and hence, constitutes a promising novel antibiotics-independent therapeutic strategy in the combat of acute campylobacteriosis.

## 1. Introduction

### 1.1. Human campylobacteriosis

*Campylobacter jejuni* and *Campylobacter coli*, both members of the family Campylobacteriaceae, are highly motile spiral-shaped Gram-negative bacteria that cause campylobacteriosis in infected humans, a severe gastroenteritis syndrome with annually approximately 500 million reported cases worldwide (Backert et al., 2017; Authority, 2021; Casalino et al., 2022). As a zoonotic pathogen, *C. jejuni* primary resides in poultry and colonizes the avian gastrointestinal tract with up to 10^8^ colony forming units (CFU) per gram feces without causing clinical symptoms (Blaser, 1997; Hermans et al., 2012). Risk factors for human infection include the consumption of undercooked contaminated chicken meat, unpasteurized milk, or surface water (Reichelt et al., 2022). Campylobacteriosis is characterized by bloody diarrhea, abdominal cramps, and systemic inflammatory responses including fever (Blaser, 1997; Burnham and Hendrixson, 2018; Lobo de Sá et al., 2021b). Although symptoms are usually self-limiting within 7-14 days, immune-compromised individuals and young children can develop an acute enterocolitis syndrome, the most severe form of campylobacteriosis (Silva et al., 2011). Depending on the infectious strain *C. jejuni* specific sialylated lipo-oligosaccharide (LOS), an essential virulence factor on the outer membrane, has been identified to trigger the hyperactivation of the intestinal innate immune system (Ellström et al., 2016; Elmi et al., 2021). Structural variations of LOS are related to highly different disease manifestations ranging from general malaise to severe enterocolitis with abdominal cramps, bloody diarrhea and fever. As a Toll-like-receptor-4 (TLR-4) ligand, *C. jejuni* LOS activates two pro-inflammatory pathways via TRIF-TRAM and nuclear factor ‘kappa-light-chain-enhancer’ of activated B-cells (NF-κB). Thus, reactive oxygen species (ROS) and several cytokines, like tumor necrosis factor-alpha (TNF-α) and interleukin 12 (IL-12), are released into the gastrointestinal tract (Cróinín and Backert, 2012). In the resulting inflammatory response neutrophilic granulocytes, macrophages and monocytes are recruited to the lamina propria and sub-epithelial tissues, which leads to accumulation of ROS inducing apoptosis, tissue destruction, and ulcerations (Young et al., 2007; Cróinín and Backert, 2012; Masanta et al., 2013). Past studies have also linked the LOS molecule to the development of post-infectious sequelae including autoimmune disorders like Guillain-Barré syndrome, reactive arthritis, and chronic inflammatory morbidities of the intestinal tract (Mortensen et al., 2009; Poropatich et al., 2010; Keithlin et al., 2014). According to the World Health Organization antibiotic resistance in *C. jejuni*, mainly against fluoroquinolones and macrolides, has become more prevalent over the past decade, posing a public health concern (Iovine, 2013). The development of novel independent strategies to combat multidrug-resistant *Campylobacter* species in terms of a One-Health approach has therefore become of high importance. In fact, research has shifted its focus to the identification of natural compounds that exhibit anti-microbial and anti-inflammatory properties without triggering resistance for future therapeutic and/or preventive applications (Kreling et al., 2020).

### 1.2. Campylobacteriosis mouse model

In the past, reliable *in vivo* models to mirror human campylobacteriosis were missing, since the murine microbiome exhibits a strong colonization resistance toward *C. jejuni* (Robertson et al., 2007). In addition, conventional mice are less responsive toward LOS, as they need 100 to 10,000-fold higher LOS concentrations to induce an inflammatory response upon *C. jejuni* infection in comparison to humans (Stahl and Vallance, 2015). To overcome these physiological limitations, secondary abiotic (SAB) interleukin-10 (IL-10) knockout mice were used. The depletion of the murine gut microbiota by antibiotic pretreatment facilitated establishment of the enteropathogens alongside the gastrointestinal tract, whereas the *il-10* gene knockout rendered mice susceptible to bacterial LOS resulting in clinical signs and immune-pathological responses upon oral *C. jejuni* infection as seen in human campylobacteriosis (Mousavi et al., 2020a). This *C. jejuni* infection and inflammation model has already been validated on numerous compounds, such as vitamins (Mousavi et al., 2020b; Lobo de Sá et al., 2021a), plant derived biomolecules (Mousavi et al., 2020c,d), essential oils (Bereswill et al., 2021a; Heimesaat et al., 2021; Mousavi et al., 2021), activated charcoal (Bereswill et al., 2021b) as well as the short chain fatty acid butyrate (Du et al., 2022), in order to assess their anti-microbial and immunomodulatory properties in acute campylobacteriosis.

### 1.3. Organic acids as potential treatment option in the combat of campylobacteriosis

Organic acids are a group of chemicals with the general structure R-COOH and have been well known for their health-beneficial effects (Dibner and Buttin, 2002). Distinct organic acids have been widely used as feed additives to enhance nutrient digestibility and growth performance. Their potential to reduce pathogen loads as an alternative, antibiotic-independent strategy has been explored *in vitro* as well (Cengiz et al., 2012; Al-Ghamdi, 2022). It is hypothesized that at low pH the undissociated form of organic acids can penetrate the bacterial membrane, thus, dissociating in the cytoplasm and causing a lethal accumulation of anions (Kovanda et al., 2019). Previous *in vitro* and *in vivo* studies assessed the potential of organic acids to counteract Gram-positive bacteria including *Clostridium perfringens* and Gram-negative enteropathogens such as *Salmonella* and *Campylobacter* species (Friedman et al., 2003). Given that *C. jejuni* induced acute enterocolitis symptoms are heavily associated with an excess secretion of pro-inflammatory mediators (Mousavi et al., 2020a), novel pharmaceutical intervention strategies should target the innate immune system and dampen ROS triggered tissue destruction. Importantly, the severity of campylobacteriosis is significantly associated with the risk for the development of post-infectious sequelae of *C. jejuni* infection (Mortensen et al., 2009) indicating that dampening of enteric inflammation constitutes a valuable measure for prevention of Guillain-Barré syndrome and other autoimmune complications of campylobacteriosis. However, data regarding potential anti-inflammatory features of organic acids in *C. jejuni* infection are rather scarce. Lately, researchers have focused on synergistic effects between organic acids or with other molecules, as it is hypothesized that the combination of distinct organic acids might enforce already existing characteristics such as antimicrobial and immunomodulatory properties (Lourenço et al., 2018; Pelyuntha et al., 2022; Szott et al., 2022). Previously, our group investigated the anti-pathogenic and anti-inflammatory effects of the short chain fatty acid (SCFA) butyrate during *C. jejuni* infection in SAB IL-10 knockout mice (Du et al., 2022). Butyrate or butyric acid is known for its anti-bacterial and immunomodulatory effects, which are mainly exhibited indirectly via immunomodulation as a histone deacetylase (HDAC) inhibitor (Ricke, 2003; Stilling et al., 2016; Du et al., 2021). Although butyrate did not lower gastrointestinal enteropathogen counts, amelioration of clinical symptoms and significant anti-inflammatory effects could be detected upon treatment of *C. jejuni* infected mice (Du et al., 2022). Building on our previous results, we now investigated potential additive and/or synergistic effects of butyrate with other organic acids, which have been reported to exhibit anti-microbial effects *in vitro*, such as phenols and medium chain fatty acids (MCFAs) (Peh et al., 2020). Phenols are plant-derived metabolites with anti-bacterial, anti-fungal, and anti-viral properties (Zapletal et al., 2022; Lima et al., 2023). A study by Friedman et al. revealed anti-microbial effects of the phenolic compound benzoic acid, a widely used food preservative, directed against several food-borne enteropathogens including *Listeria monocytogenes*, *Escherichia coli, Salmonella enterica*, and *C. jejuni* (Friedman et al., 2003). MCFAs have gained attention for their anti-microbial activity against the gastric pathogen *Helicobacter pylori* (Petschow et al., 1996). Caprylic acid constitutes a saturated C8 fatty acid and has also been shown to reduce intestinal *C. jejuni* loads in chicken (Solis de Los Santos et al., 2008). Therefore, butyric acid was chosen to be combined with benzoic acid as the phenolic compound and caprylic acid as the MCFA. Furthermore, we decided to include sorbic acid in our treatment regimens given its synergistic anti-microbial, including anti-*C. jejuni* directed, effects in combination with other organic acids, as shown for formic acid (Peh et al., 2020). Furthermore, respective four organic acids were chosen under the premise that all substances are listed as authorized feed additives by the European Union and thus, can be directly transferred into clinical use. In addition, the applied organic acids exhibited relatively low minimal inhibitory concentrations (MICs) against *C. jejuni* when compared to other compounds within the same chemical group (Peh et al., 2020; European Commission, Directorate-General for Health and Food Safety, 2021). Practical aspects such as water solubility and low production costs have been taken into consideration as well (Bialkowski et al., 2022).

### 1.4. Objective

Thus, the objective of the current study was to assess disease alleviating effects of therapeutic application of benzoic acid, butyric acid, caprylic acid, and sorbic acid individually and in combination to SAB *C. jejuni* infected IL-10^–/–^ mice due to potential anti-pathogenic and/or anti-inflammatory immune responses.

## 2. Materials and methods

### 2.1. Ethics statement and animal welfare

All protocols followed the guidelines stated in the directive 2010/63/EU (requirements for animal protection used for scientific purposes in the European Union) and were authorized by the ethical committee in Berlin (“Landesamt für Gesundheit und Soziales”, Berlin, Germany; No. G0104/19). To ensure animal welfare the clinical status of each mouse was surveyed every day. In addition, mice were housed in a maximum of three animals per cage in a semi-barrier experimental setup (cycle of 12 h light/12 h dark, 22–24°C, 55 ± 15% humidity). Feed consisted of standard chow (ssniff R/M-H, V1534-300, Sniff, Soest, Germany) and autoclaved tap water was accessible *ad libitum*.

### 2.2. Generation of secondary abiotic IL-10^–/–^ mice

To eradicate the murine gut microbiota, 3-week-old IL-10^–/–^ mice were administered an antibiotic cocktail (ABx) for 8 weeks, which was composed of ciprofloxacin (200 mg/L; Fresenius Kabi, Bad Homburg, Germany), imipenem (250 mg/L; Fresenius Kabi, Bad Homburg, Germany), vancomycin (500 mg/L; Hikma Pharmaceuticals, London, UK), metronidazole (1 g/L; B. Braun, Melsungen, Germany) and ampicillin plus sulbactam (2 g/L; Dr. Friedrich Eberth Arzneimittel, Ursensollen, Germany) dissolved in autoclaved tap water. To prevent cross-contamination all animals were handled aseptically in sterile cages with filter tops. Two days prior to the *C. jejuni* infection, ABx was replaced with sterile drinking water to induce antibiotic washout. To ensure the total absence of murine microbes, fecal samples were collected during the antibiotic pretreatment period as well as immediately before pathogen infection and cultured in enrichment broths as described in detail earlier (Heimesaat et al., 2006).

### 2.3. *C. jejuni* infection

Prior to the infection *C. jejuni* strain 81-176 was derived from frozen stocks, inoculated on karmali agar plates (Oxoid, Wesel, Germany) and grown at 37°C under microaerophilic conditions (CampyGen gas packs, Oxoid, Wesel, Germany). After 2 days of incubation one fluently grown karmali plate was chosen for harvest and incorporated in 5 mL sterile phosphate-buffered saline (PBS; Thermo Fisher Scientific, Waltham, MA, USA). This resulted in an optical density of 0.6 at 600 nm wavelength with a yield of 10^9^ viable bacterial cells. On days 0 and 1, a volume of 0.3 mL of the *C. jejuni* suspension was used to gavage each SAB IL-10^–/–^ mouse. The yield of 10^9^ colony-forming units (CFU) was respectively confirmed by serial dilutions and culture of the bacterial suspensions on solid culture media as described recently (Du et al., 2022).

### 2.4. Treatment with organic acids

The animals had access to the treatment solutions from day 2 until day 6 p.i., whereas the placebo group, consisting of age- and sex-matched litter mate mice, received autoclaved tap water instead. Benzoic acid (Sigma-Aldrich, München, Germany), sodium butyrate (Sigma-Aldrich, München, Germany), sodium caprylate (Sigma-Aldrich, München, Germany), and sorbic acid (Sigma-Aldrich, München, Germany) were dissolved each in autoclaved tap water and sterile filtered before being applied as drinking solutions *ad libitum*. Due to the low acceptance of mice toward high acidic liquids, 1M NaOH was added to the benzoic acid, sorbic acid, and combination treatment to reach a neutral pH (i.e., 7.3 ± 0.2). Butyrate and caprylate drinking solutions already exhibited a neutral pH after suspension in water. The daily dosages and final concentrations based on previously assessed MICs are minimal inhibitory concentrations (MICs) are summarized in Table 1.

**TABLE 1:** Minimum inhibitory concentrations (MICs) and concentrations of applied drinking solutions for benzoic acid, butyric acid, caprylic acid, and sorbic acid (alone and all combined).

	MIC at pH 7.3	Concentration of organic acids solutions	Daily dosage
Benzoic acid	977 mg/L (Peh et al., 2020)	3,900 mg/L	781.5 mg/kg/day 19.53 mg/mouse/day
Butyric acid	1761 mg/L (Du et al., 2022)	22,000 mg/L	4,400 mg/kg/day 110 mg/mouse/day
Caprylic acid	288.4 mg/L (Peh et al., 2020)	1,150 mg/L	230 mg/kg/day 5.75 mg/mouse/day
Sorbic acid	718 mg/L (Peh et al., 2020)	897 mg/L	179.2 mg/kg/day 4.48 mg/mouse/day
Combination	256 mg/L	27,947 mg/L	5,590 mg/kg/day 139.7 mg/mouse/day

### 2.5. Clinical outcome

During the experimental period, clinical symptoms and wasting aspects (0: normal; 1: ruffled fur; 2: reduced locomotion; 3: isolation; 4: severely impaired locomotion and prefinal aspect) were evaluated for every individual mouse daily. In addition, fecal samples were examined for diarrhea (0: normal feces; 2: pasty consistency; 4: liquid consistency) and tested for the absence or presence (microscopic or macroscopic) of blood (0: normal feces, 2: positive Guaiac-based fecal occult blood testing (Haemoccult, Beckman Coulter/PCD, Krefeld, Germany), 4: visible appearance of blood). The single scores of wasting, diarrhea, and fecal blood were pooled to a cumulative score to describe the overall clinical outcome.

### 2.6. Sample processing

On day 6 p.i. mice were sacrificed by gas asphyxiation using carbon dioxide and dissected under aseptic conditions. *Ex vivo* biopsies were taken from colon and mesenteric lymph nodes (MLN) for further pro-inflammatory mediator measurements. In addition, colonic parts were derived for quantitative *in situ* immunohistochemical staining of apoptotic and immune cells, as previously reported (Heimesaat et al., 2018). Gastrointestinal luminal samples of the colon, ileum, duodenum, and stomach were collected for cultural analysis of *C. jejuni* as described earlier (Du et al., 2022). In brief, serial dilutions of luminal samples (in sterile PBS; Thermo Fisher Scientific, Waltham, MA, USA) were streaked onto karmali agar plates (Oxoid, Wesel, Germany) and incubated for 48 h at 37°C under microaerophilic conditions (CampyGen gas packs, Oxoid, Wesel, Germany). The CFU counts were normalized to the weights of the fecal sample. The detection limit of *C. jejuni* was 100 CFU per g.

### 2.7. Immunohistochemical staining

*Ex vivo* colonic samples were immediately fixed in 5% formalin during the dissection process and embedded in paraffin. Sections of 5 μm were then examined under light microscopy (100 × magnification) after staining with hematoxylin and eosin (H&E) and applying an established histopathological scoring system as previously described (Heimesaat et al., 2018). To detect apoptotic cells, neutrophils, macrophages and monocytes, regulatory T cells as well as T and B lymphocytes, the paraffin sections were stained with primary antibodies against caspase-3 (Asp175, Cell Signaling, Beverly, MA, USA, 1:200), MPO7 (No. A0398, Dako, Glostrup, Denmark, 1:500), F4/80 (No. 14-4801, clone BM8, eBioscience, San Diego, CA, USA, 1:50), Foxp3 (clone FJK-165, no. 14-5773, eBioscience, 1:100), CD3 (No. N1580, Dako, Glostrup, Denmark, 1:10), and B220 (No. 14-0452-81, eBioscience, San Diego, CA, USA, 1:200), respectively.

### 2.8. Measurements of pro-inflammatory mediators

*Ex vivo* colonic biopsies of approximately 1 cm^2^ from the colon were longitudinally cut and washed in PBS. Colon tissue and samples of MLN (3 nodes in total) were cultured for 18 h at 37^°^C in 24-flat-bottom well culture plates (Thermo Fisher Scientific, Waltham, MA, USA) containing 500 μL serum-free RPMI 1640 medium (Thermo Fisher Scientific, Waltham, MA, USA), plus penicillin (100 U/mL; Biochrom, Berlin, Germany) and streptomycin (100 μg/mL; Biochrom, Berlin, Germany). Following the manufacturers’ protocol for the Mouse Inflammation Cytometric Bead Array (CBA; BD Biosciences, Heidelberg, Germany) on a BD FACSCanto II flow cytometer (BD Biosciences, Heidelberg, Germany), respective culture supernatant samples were tested for interleukin-6 (IL-6), interferon-γ (IFN-γ), tumor necrosis factor-alpha (TNF-α), and monocyte chemoattractant protein-1 (MCP-1).

### 2.9. Data analysis

Data were pooled from three independent experiments, using Prism (version 9, GraphPad, San Diego, CA, USA) to analyze medians and significance levels [two-sided probability (*p*) values ≤ 0.05] applying Student’s *t*-test and the Mann–Whitney test for pairwise comparisons of normally and not normally distributed data, respectively. For multiple comparisons, the one-sided ANOVA with Tukey’s post-correction was used for normally distributed data, whereas the Kruskal–Wallis test with Dunn’s post-correction was applied for not normally distributed data.

## 3. Results

### 3.1. Kinetic survey and gastrointestinal pathogen loads following organic acid treatment of *C. jejuni* infected IL-10^–/–^ mice

**FIGURE 1:**
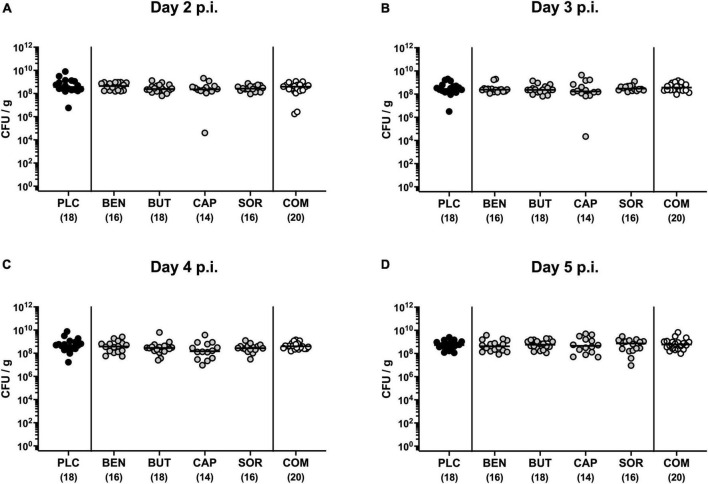
Kinetic survey of pathogen loads post-infection upon single and combined organic acid treatment of *C. jejuni* infected IL-10^–/–^ mice. Secondary abiotic (SAB) IL-10^–/–^ mice were perorally infected with *C. jejuni* strain 81-176 on days 0 and 1. Starting on day 2 post-infection (p.i.), the mice were treated with benzoic acid (BEN), butyric acid (BUT), caprylic acid (CAP), sorbic acid (SOR), or a combination (COM) of respective organic acids via the drinking water for 4 days. The placebo (PLC) group only received autoclaved tap water. The pathogen loads were quantified on **(A)** day 2, **(B)** day 3, **(C)** day 4, and **(D)** day 5 p.i. by culture (colony forming units per gram; CFU/g). Medians (black bars) and total mice numbers (in parentheses) are indicated. Data were pooled from three independent experiments.

**FIGURE 2:**
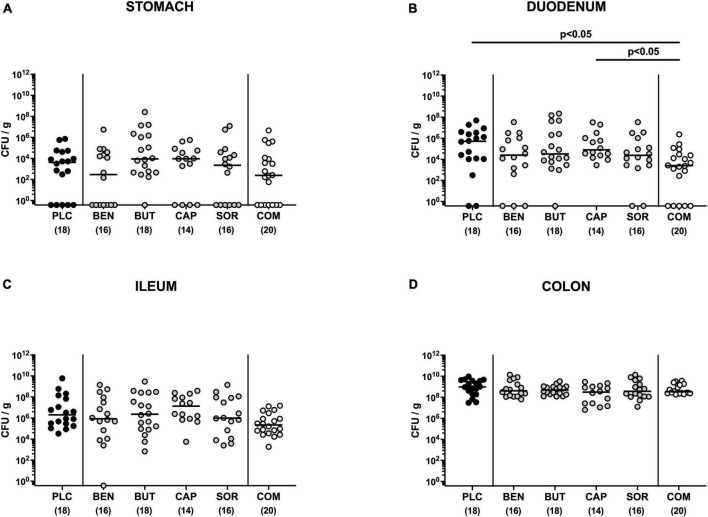
Gastrointestinal pathogen loads following single and combined organic acid treatment of infected mice. Starting on day 2 post-infection, the mice were treated with benzoic acid (BEN), butyric acid (BUT), caprylic acid (CAP), sorbic acid (SOR), or a combination (COM) of respective organic acids via the drinking water for 4 days. The placebo (PLC) group only received autoclaved tap water. On day 6 post-infection, *C. jejuni* loads were determined in luminal samples taken from the **(A)** stomach, **(B)** duodenum, **(C)** ileum, and **(D)** colon by culture (colony forming units per gram; CFU/g). Medians (black bars), significance levels (*p* values) calculated by the ANOVA test with Tukey’s post-correction or by the Kruskal–Wallis test and Dunn’s post-correction, and the total mice numbers (in parentheses) are given. Data were pooled from three independent experiments.

First, we tested whether treatment with respective organic acids had an anti-microbial effect on *C. jejuni* in infected mice. To address this, mice were challenged with 10^9^ viable *C. jejuni* strain 81-176 on days 0 and 1 by gavage. Starting from day 2 p.i., SAB IL-10^–/–^ mice were subjected to the organic acids either alone or all in combination via the drinking water. Daily cultural analysis of fecal samples revealed that the application of respective organic acids neither alone nor in combination affected intestinal colonization properties of *C. jejuni* as indicated by comparable fecal pathogen loads in treated and placebo control mice [not significant (n.s.); Figure 1]. Upon sacrifice we further determined pathogenic burdens in distinct gastrointestinal luminal sites. On day 6 p.i., approximately two orders of magnitude lower *C. jejuni* numbers were detected in the duodenum of mice from the organic acid combination treatment group as compared to placebo counterparts (*p* < 0.05; Figure 2B), whereas in the stomach, ileum, and colon *C. jejuni* loads were comparable in respective cohorts (n.s.; Figures 2A, C, D). Hence, only the combination organic acid treatment lowered pathogen loads in the duodenum of *C. jejuni* infected mice.

### 3.2. Clinical outcome following organic acid treatment of mice with acute campylobacteriosis

**FIGURE 3:**
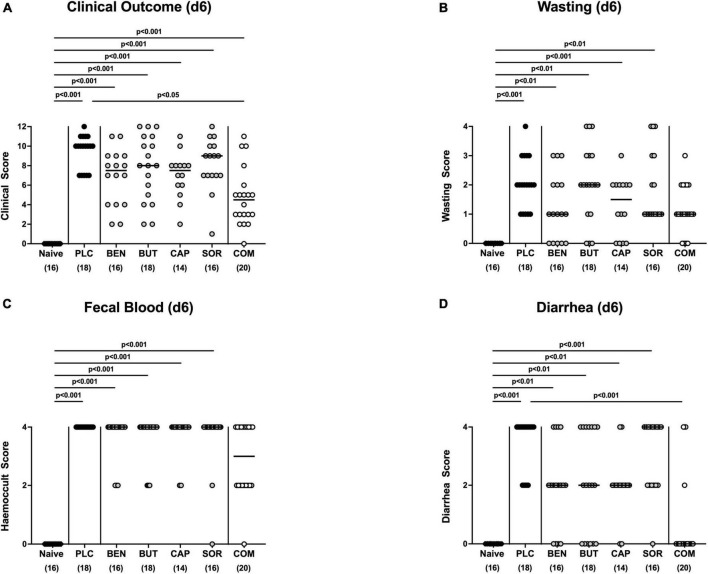
Clinical outcome upon single and combined organic acid treatment on day 6 post-infection. Infected mice received a treatment with benzoic acid (BEN), butyric acid (BUT), caprylic acid (CAP), sorbic acid (SOR), or a combination of all acids (COM), whereas the placebo (PLC) group received autoclaved tap water only. On day 6 (d6) post-infection, **(A)** the clinical outcome was assessed in each mouse by standardized clinical scores evaluating in particular **(B)** wasting, **(C)** fecal blood, and **(D)** stool consistency (diarrhea). Naive mice served as untreated and uninfected controls. Medians (black bars), significance levels (*p* values) calculated by the ANOVA test with Tukey’s post-correction or by the Kruskal–Wallis test and Dunn’s post-correction and the total mice numbers (in parentheses) are given. Data were pooled from three independent experiments.

At the end of the observation period, namely day 6 p.i., mice from the placebo cohort suffered from severe campylobacteriosis and showed symptoms such as wasting, fecal blood, and diarrhea characteristic for acute enterocolitis (*p* < 0.01–0.001 vs. naive; Figure 3). Remarkably, mice from the combination organic acid treatment group were clinically far less compromised on day 6 p.i. as indicated by lower scores for the overall clinical outcome (*p* < 0.05 vs. placebo; Figure 3A) and for diarrheal symptoms (*p* < 0.001 vs. placebo; Figure 3D). Of note, 85% of mice treated with the combination organic acid regimen did not suffer from diarrhea at all whereas all placebo control animals presented with diarrheal symptoms (Figure 3D). Furthermore, whereas on day 6 p.i. clinical scores for wasting, fecal blood and diarrhea were elevated in all placebo and mono-treated mice (*p* < 0.01–0.001 versus naive; Figures 3B–D), organic acid combination treated animals displayed clinical scores for respective parameters that did not differ from naive controls (n.s.; Figures 3B–D). Hence, combination organic acid treatment of *C. jejuni* infected IL-10^–/–^ mice improved the overall clinical outcome of acute campylobacteriosis and particularly alleviated diarrheal symptoms in infected mice.

### 3.3. Macroscopic and microscopic inflammatory sequelae following organic acid treatment

**FIGURE 4:**
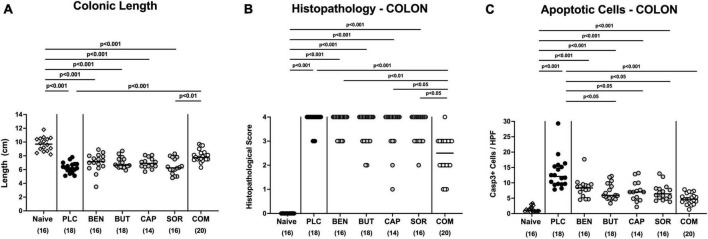
Macroscopic and microscopic inflammatory responses in the colon following single and combined organic acid treatment. *C. jejuni* infected mice were treated with benzoic acid (BEN), butyric acid (BUT), caprylic acid (CAP), sorbic acid (SOR), a combination of all acids (COM) or received placebo (PLC). On day 6 post-infection, **(A)** the colonic lengths were measured and **(B)** the histopathological changes in hematoxylin and eosin-stained *ex vivo* colonic biopsies were quantified by standardized histopathology scores. **(C)** Furthermore, the average numbers of apoptotic colonic epithelial cells (positive for caspase3, Casp3+) were determined microscopically from six high power fields (HPF, 400× magnification) per mouse applying *in situ* immunohistochemical analysis of large intestinal paraffin sections. Naive mice were used as negative controls. Medians (black bars), significance levels (*p* values) calculated by the ANOVA test with Tukey’s post-correction or by the Kruskal–Wallis test and Dunn’s post-correction, and the total mice numbers (in parentheses) are given. Data were pooled from three independent experiments.

**FIGURE 5:**
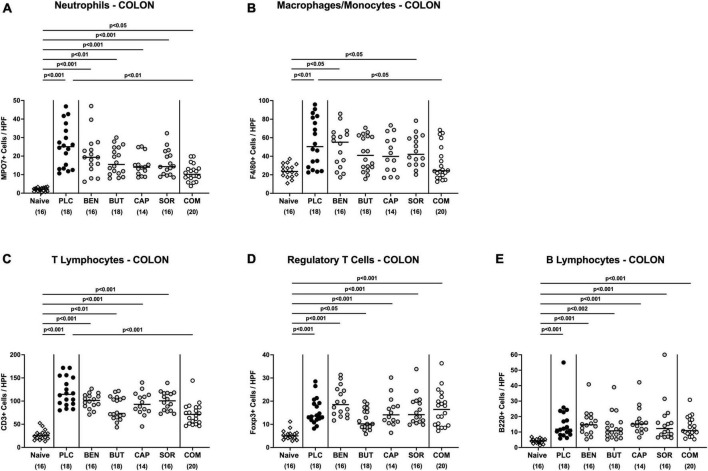
Colonic immune cell responses upon single and combined organic acid treatment. Starting on day 2 post-infection, *C. jejuni* infected mice received a 4-day treatment with benzoic acid (BEN), butyric acid (BUT), caprylic acid (CAP), sorbic acid (SOR), a combination of all acids (COM) or placebo (PLC). On day 6 post-infection, the average numbers of **(A)** neutrophils (positive for MPO7), **(B)** macrophages and monocytes (positive for F4/80), **(C)** T lymphocytes (positive for CD3), **(D)** regulatory T cells (positive for Foxp3), and **(E)** B lymphocytes (positive for B220) in the colonic mucosa and lamina propria from six high power fields (HPF, 400× magnification) per animal were assessed in immunohistochemically stained colonic paraffin sections. Naive mice served as uninfected and untreated controls. Medians (black bars), significance levels (*p* values) calculated by the ANOVA test with Tukey’s post-correction or by the Kruskal–Wallis test and Dunn’s post-correction, and the total mice numbers (in parentheses) are given. Data were pooled from three independent experiments.

We next measured the colonic length of each mouse upon necropsy, since intestinal inflammation is accompanied by shortening of the affected gut compartments (Heimesaat et al., 2006; Bereswill et al., 2011). At day 6 p.i. the colonic length of organic acid combination treated mice remained virtually unaffected, whereas the animals from the individual organic acids and placebo groups displayed shorter colonic lengths after *C. jejuni* infection (*p* < 0.001; Figure 4A). We further quantitively surveyed the severity of colonic inflammation on a microscopic level, applying a histopathological scoring system on H&E stained *ex vivo* colonic biopsies. *C. jejuni* infected control mice displayed severe histopathological changes on day 6 p.i., which were, however, significantly less pronounced in organic acid combination treated mice (*p* < 0.001; Figure 4B). In addition, we assessed *C. jejuni* induced apoptotic cell responses in colonic samples by *in situ* immunohistochemistry. At day 6 p.i. organic acid combination treated mice exhibited fewer caspase 3 + apoptotic cells in their colonic epithelia in comparison to the control cohort (*p* < 0.001; Figure 4C). When applied individually, butyric acid, caprylic acid, and sorbic acid also led to a reduction of apoptotic cells in the colonic tissue (*p* < 0.05–0,001; Figure 4C). To further explore the immunomodulatory properties of organic acids, we quantified distinct immune cell populations in the mucosa and lamina propria of the colon using immunohistochemistry. Upon sacrifice, colonic numbers of neutrophils, macrophages, monocytes and T lymphocytes were significantly lower in the organic acid combination than in placebo treated mice (*p* < 0.05–0.001; Figures 5A–C). In case of F4/80 + macrophages and monocytes, infected mice from the butyrate, caprylate and combination cohorts exhibited basal colonic cells counts (n.s. vs. naive; Figure 5B). The *C. jejuni* induced increases of regulatory T cells and B lymphocytes within colonic biopsies were, however, comparable in all cohorts (*p* < 0.05–0.001 versus naive; Figures 5D, E). Hence, the organic acid combination treatment resulted in lower macroscopic and microscopic inflammatory immune responses in *C. jejuni* infected IL-10^–/–^ mice.

### 3.4. Intestinal pro-inflammatory mediators following organic acid treatment

**FIGURE 6:**
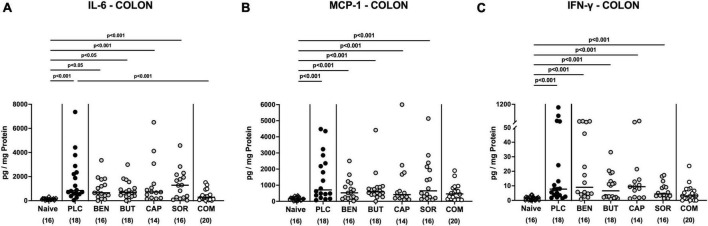
Colonic pro-inflammatory mediator responses upon single and combined organic acid treatment. Infected mice received benzoic acid (BEN), butyric acid (BUT), caprylic acid (CAP), sorbic acid (SOR), a combination of all acids (COM) or placebo (PLC). On day 6 post-infection, pro-inflammatory mediators including **(A)** IL-6, **(B)** MCP-1, and **(C)** IFN-γ were measured in supernatants of *ex vivo* biopsies derived from the colon. Naive mice served as negative controls. Medians (black bars), significance levels (*p* values) calculated by the ANOVA test with Tukey’s post-correction or by the Kruskal–Wallis test and Dunn’s post-correction, and the total mice numbers (in parentheses) are given. Data were pooled from three independent experiments.

**FIGURE 7:**
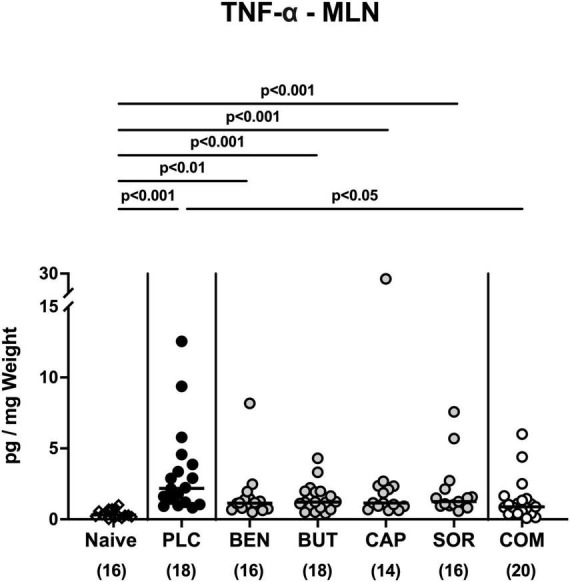
Pro-inflammatory mediator secretion in mesenteric lymph nodes upon single and combined organic acid treatment. Infected mice were perorally treated with benzoic acid (BEN), butyric acid (BUT), caprylic acid (CAP), sorbic acid (SOR), or a combination of all acids (COM). The placebo (PLC) group only received autoclaved tap water. On day 6 post-infection, TNF-α concentrations were assessed in supernatants of *ex vivo* biopsies derived from the mesenteric lymph nodes (MLN). Medians (black bars), significance levels (*p* values) calculated by the ANOVA test with Tukey’s post-correction or by the Kruskal-Wallis test and Dunn’s post-correction, and the total mice numbers (in parentheses) are given. Data were pooled from three independent experiments.

We further surveyed the anti-inflammatory properties of the applied organic acids, and assessed pro-inflammatory mediator secretion in the distinct intestinal compartments. Six days following *C. jejuni* infection elevated IL-6, MCP-1, and IFN-γ concentrations were measured in the colonic tissues of mice treated with placebo and respective organic acids alone (*p* < 0.05–0.001 vs. naive; Figure 6), whereas mice from the organic acid combination cohort exhibited colonic mediator concentrations that were comparable to basal levels (n.s. vs. naive; Figure 6). Furthermore, colonic IL-6 concentrations in organic acid combination treated mice were significantly lower when compared to placebo counterparts on day 6 p.i. (*p* < 0.001; Figure 6A). In support, *C. jejuni* infection resulted in increased TNF-α concentrations measured in the MLN of mice treated with placebo or respective organic acids alone (*p* < 0.01–0.001 vs. naive; Figure 7), which held, however, not true for the combination cohort exhibiting basal TNF-α levels in their MLN (n.s. vs. naive; *p* < 0.05 vs. placebo; Figure 7). Hence, treatment with the organic acid combination dampened *C. jejuni* induced pro-inflammatory mediator secretion in the intestinal tract to naive levels.

### 3.5. Systemic pro-inflammatory effects following organic acid treatment

**FIGURE 8:**
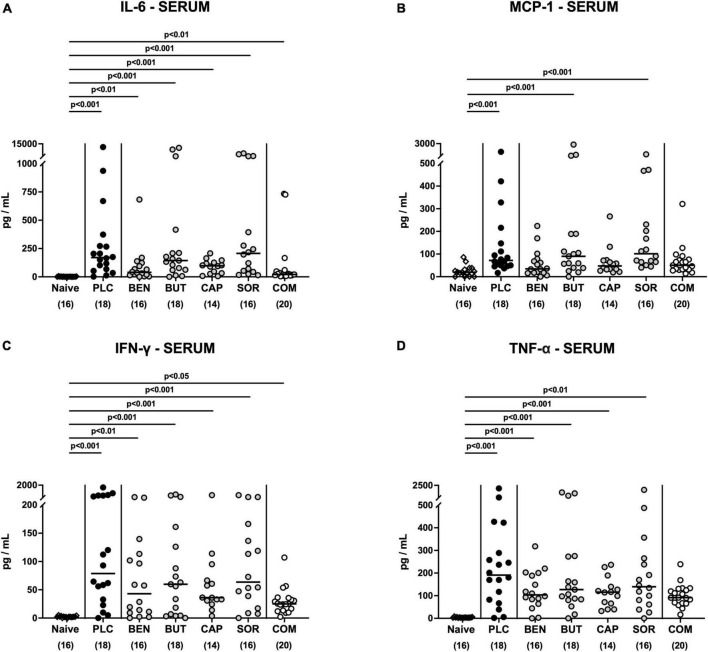
Systemic pro-inflammatory mediator secretion following organic acid treatment. From day 2 until day 6 post-infection, the mice received an oral treatment with benzoic acid (BEN), butyric acid (BUT), caprylic acid (CAP), sorbic acid (SOR), a combination of all acids (COM) or placebo (PLC), whereas naive mice served as untreated and uninfected controls. **(A)** IL-6, **(B)** MCP-1, **(C)** IFN-γ, and **(D)** TNF-α concentrations were measured in serum samples taken on day 6 post-infection. Medians (black bars), significance levels (*p* values) calculated by the ANOVA test with Tukey’s post-correction or by the Kruskal–Wallis test and Dunn’s post-correction and the total mice numbers (in parentheses) are given. Data were pooled from three independent experiments.

We then asked if the immunomodulatory properties of organic acids were restricted to the intestinal tract or could also be observed systemically. Irrespective of the treatment regimen, elevated IL-6 and IFN-γ concentrations were measured in serum samples taken on day 6 p.i. (*p* < 0.05–0.001; Figures 8A, C). Remarkably, basal MCP-1 and TNF-α serum concentrations were assessed in *C. jejuni* infected mice from the combination treatment cohort (n.s. vs. naive; Figures 8B, D), which was also true for MCP-1 concentrations measured in serum samples taken from benzoic acid and caprylic acid treated mice on day 6 p.i. (n.s. vs. naive; Figure 8B). Hence, the anti-inflammatory effects of oral benzoate, butyrate, caprylate and sorbate in combination were not only effective in the intestinal tract, but could also be assessed systemically in *C. jejuni* infected IL-10^–/–^ mice.

## 4. Discussion

Our present intervention study is the first to evaluate the disease-ameliorating effects of benzoic acid, butyric acid, caprylic acid, and sorbic acid either alone or in combination for the treatment of acute campylobacteriosis in a murine infection model. The anti-inflammatory effects of the organic acid combination treatment were proven in the intestinal tract and remarkably, also systemically. Peroral application of respective organic acids all in combination to *C. jejuni* infected SAB IL-10^–/–^ mice resulted in: (i) improved clinical outcome and particularly in pronounced anti-diarrheal effects on day 6 p.i.; (ii) less distinct macroscopic signs of inflammation such as colonic shrinkage; (iii) dampened microscopic inflammatory sequelae such as less pronounced colonic histopathology and apoptosis; (iv) less large intestinal accumulation of distinct immune cell populations such as neutrophils, macrophages, monocytes, and T lymphocytes; and furthermore, (v) dampened pro-inflammatory cytokine secretion in the colon and MLN and (vi) even the serum.

It is noteworthy that the combination organic acid treatment induced health-beneficial properties without lowering the pathogen loads in the colon, ileum, and stomach, implying that a reduction of the duodenal large intestinal *C. jejuni* load might not be of biological relevance for the observed therapeutic effects. The combined organic acid treatment significantly prevented from diarrhea, as 85% of infected mice treated with the combination regimen did not suffer from any changes in stool consistency at all. The determination of natural compounds like organic acid with high anti-inflammatory properties and rather low anti-bacterial effects might even be beneficial regarding the prevention of gut dysbiosis. Although previous *in vitro* results revealed anti-*C. jejuni* directed properties of butyric acid, benzoic acid, caprylic acid, and sorbic acid individually or applied with other compounds (Peh et al., 2020), the anti-pathogenic effects in our present study were limited to the duodenum. It was proposed that MCFAs may trigger membrane damage and lead to an influx of SCFAs (Peh et al., 2020). We hypothesize that the virtually absent anti-microbial effects in the distal intestines in our study might be due to the buffered acidification of organic acids in living organisms, by colonic mucus and other intestinal contents. In addition, premature absorption effects by enterocytes cannot be excluded. Butyrate, for instance, can also be utilized as an energy source (Luethy et al., 2017). Most importantly, the combined organic acid treatment of acute enterocolitis in *C. jejuni* infected mice resulted in reduced macroscopic and microscopic pro-inflammatory responses. The improved clinical outcome on day 6 p.i. could be attributed to dampened intestinal inflammation and innate immune response, as implied by lower histopathological scores in organic acid combination treated animals. This was further confirmed by reduced accumulation of neutrophils, macrophages, monocytes, and T lymphocytes in the large intestine in comparison to the placebo group. The anti-inflammatory effect of combination treated mice is further supported by the decreased secretion of pro-inflammatory cytokines including IL-6, MCP-1, and IFN-γ in the colonic tissue. In addition, lower TNF-α concentrations were assessed in the MLN. This is in line with a previous study that reported synergistic effects between organic acids in the prevention of intestinal inflammation by lowering the production of IL-6 and TNF-α after infection with enterohemorrhagic *Escherichia coli* in mice (Wang et al., 2018b). Remarkably, the combination treatment regimen could dampen systemic pro-inflammatory mediator secretion to basal levels as indicated by TNF-α and MCP-1 and serum concentrations measured on day 6 p.i. that did not differ from uninfected controls.

Although the exact molecular mechanisms for potential additive or synergistic immunomodulatory effects of the used organic acids remain unclear, butyric and caprylic acid, for instance, both act as histone deacetylase inhibitors and induce host defense peptides and may have mutually reinforced their abilities (Wang et al., 2018a). Another possible mechanism underlying the anti-inflammatory effects of combined organic acids may involve TLR-4 antagonism. *C. jejuni* induced diarrhea has been shown to be triggered by pathogen specific lipo-polysaccharide (LPS), a common microbial surface structure among Gram-negatives, which, in turn, leads to intestinal immunopathological processes by TLR-4 activation (Haag et al., 2012; Mousavi et al., 2020a). Recently, it has been revealed that sodium butyrate supplementation reduces LPS-induced murine diarrhea by downregulated expression of genes encoding for ZO-1 and NLRP3 and upregulated expression of genes encoding for occludin, claudin, and caspase-1 (Chen et al., 2022). In addition, caprylic acid has been shown to exert its anti-inflammatory effects by suppressing TLR-4 and NF-κB signaling pathways (Zhang et al., 2019). Interestingly, macrophage inflammatory responses have also been shown to be regulated by TLR-4 signaling (Swanson et al., 2020). This is in line with our present study, since clinical symptoms such as diarrhea, as well as apoptotic cells within the colonic tissue were significantly reduced in butyric, caprylic acid and especially organic acid combination treated mice. Although singularly applied organic acids did not dampen innate immune cell accumulation in the infected colon in comparison to controls, our combination organic acid treatment led to a significant reduction in colonic numbers of neutrophils, macrophages and monocytes of the investigated organic acids. We therefore hypothesize that the combinatory effects of the here applied organic acids occurred by downregulation of TLR-4 and NF-κB pathway signaling responses.

Overall, the applied organic acids are generally considered safe and have widely been used as feed additives and performance enhancers in animal farming with no significant side effects being reported so far (Nguyen et al., 2020). In fact, caprylic acid constitutes an anti-microbial compound in breast milk (Gardner et al., 2017). Although organic acids have already been validated as easy to administer with low costs, optimization for practical use might still be necessary. For instance, the strong taste and unpleasant smell of butyric acids might interfere with compliance to ingest infused feed and drinking. Therefore, esterified derivatives or coated pro-drugs might pose as a more acceptable alternative for clinical trials. In addition, ester forms are naturally more stable and can even escape gastric digestion before reaching the small intestine (Friedman et al., 2003).

## 5. Conclusion

The results obtained from our preclinical intervention study display a potential basis for the development of novel therapeutic strategies against human campylobacteriosis. Due to the strong anti-diarrheal effects of the organic acid combination treatment, other foodborne diseases should be evaluated as well for respective disease ameliorating effects. Furthermore, the dampening of the pathogen-induced pro-inflammatory immune responses during acute campylobacteriosis might contribute to the prevention of post-infectious autoimmune sequelae including Guillain-Barré syndrome.

## Data availability statement

The original contributions presented in this study are included in the article/supplementary material, further inquiries can be directed to the corresponding author.

## Ethics statement

This animal study was reviewed and approved by the Landesamt für Gesundheit und Soziales, Berlin, Germany.

## Author contributions

KD performed experiments, analyzed data, and wrote the manuscript. MF, AB, and SS performed experiments and analyzed data. SM analyzed data and critically discussed results. SB provided advice on experimental design, critically discussed results, and edited the manuscript. MH designed and performed experiments, analyzed data, and wrote the manuscript. All authors read and agreed to the published version of the manuscript.
